# Characterization of protein redox dynamics induced during light-to-dark transitions and nutrient limitation in cyanobacteria

**DOI:** 10.3389/fmicb.2014.00325

**Published:** 2014-07-03

**Authors:** Charles Ansong, Natalie C. Sadler, Eric A. Hill, Michael P. Lewis, Erika M. Zink, Richard D. Smith, Alexander S. Beliaev, Allan E. Konopka, Aaron T. Wright

**Affiliations:** Biological Sciences Division, Pacific Northwest National LaboratoryRichland, WA, USA

**Keywords:** cyanobacteria, nutrient limitation, light-dark transition, activity-based protein profiling, chemical proteomics

## Abstract

Protein redox chemistry constitutes a major void in knowledge pertaining to photoautotrophic system regulation and signaling processes. We have employed a chemical biology approach to analyze redox sensitive proteins in live *Synechococcus* sp. PCC 7002 cells in both light and dark periods, and to understand how cellular redox balance is disrupted during nutrient perturbation. The present work identified 300 putative redox-sensitive proteins that are involved in the generation of reductant, macromolecule synthesis, and carbon flux through central metabolic pathways, and may be involved in cell signaling and response mechanisms. Furthermore, our research suggests that dynamic redox changes in response to specific nutrient limitations, including carbon and nitrogen limitations, contribute to the regulatory changes driven by a shift from light to dark. Taken together, these results contribute to a high-level understanding of post-translational mechanisms regulating flux distributions and suggest potential metabolic engineering targets for redirecting carbon toward biofuel precursors.

## Introduction

Cyanobacteria are photoautotrophic organisms equipped to convert solar irradiance into chemical energy, thereby generating reducing equivalents and ATP for CO_2_ fixation and biosynthesis of macromolecules. This highly efficient system for producing a broad variety of organic compounds makes these photoautotrophs important organisms for biofuel production technology (Ducat et al., 2011). Successful exploitation of cyanobacteria will require detailed knowledge of the regulation of cellular subsystems and networks involved in electron transport, reductant partitioning, and energy storage pathways.

The regulatory mechanisms in cyanobacteria evolved under the selective pressures they experience in their natural habitats. Growth in these habitats can be limited by availability of resources [such as light, carbon (C), nitrogen (N) or phosphorus] (Schwarz and Forchhammer, 2005), or by exposure to excessively high light intensities that can lead to photooxidative damage to the photosynthetic apparatus (Lee and Rhee, 1999). Regulation can operate to quickly recalibrate metabolic activity in order to efficiently utilize available resources (Schwarz and Grossman, 1998; Ludwig and Bryant, 2011).

As in other microorganisms, regulation at the transcriptional level is an important mechanism for adapting to changes in light and nutrient availability (Gill et al., 2002; Wang et al., 2004; Ludwig and Bryant, 2011, 2012). In addition to this relatively slow coarse control in regulating the types and amounts of protein products, both non-covalent and covalent post-translational changes in proteins can alter their activities. However, the biochemical mechanisms employed to coordinate metabolic processes are poorly understood, particularly in cyanobacteria. Because a centrally important part of cyanobacterial metabolism is the generation of reductant via photosynthesis and its consumption in the Calvin-Benson cycle and other biosynthetic pathways, the rapid sensing of redox status and regulation to balance reductant production and consumption rates is critical. Furthermore, an understanding of how redox modification of proteins occurs under physiologically relevant nutrient and light limitation could be exploited to direct metabolism to molecules useful as biofuels.

We have recently described an *in vivo* chemical profiling strategy that allowed identification of 176 proteins undergoing dynamic redox change in response to nutrient (C) limitation and subsequent replenishment in the photoautotrophic cyanobacterium *Synechococcus* sp. PCC 7002 (hereafter, *Synechococcus* 7002) (Sadler et al., 2014). Herein, we employ this chemoproteomic approach to identify redox responsive proteins in *Synechococcus* 7002 in different physiological contexts. We have examined the effect of a shift from light to dark upon the capacity to alter the reduction level of specific proteins under nutrient (C and N) limiting, steady-state growth conditions. The present work identifies over 350 putative redox-sensitive proteins that are involved in the generation of reductants, macromolecule synthesis, and C flux through central metabolic pathways, and may be involved in cell signaling and response mechanisms. Furthermore, our results suggest that dynamic redox changes in response to specific nutrient limitations amplify the redox changes being driven solely by light to dark transitions.

## Materials and methods

### Bioreactor

A New Brunswick Scientific BioFlo 310 bioreactor with a custom 7.5 L vessel was used with a 5.5 L working volume maintained at 30°C with a 250 rpm agitation rate. The pH was held at 7.5 with controlled input of 2 M NaOH or 2 M HCl as needed. Bubbling was done with pure nitrogen at 0.75 L/min. Continuous culture was done with a dilution rate of 0.1/h, which is 9.2 mL/min media delivery rate in a 5.5 L working volume. Dilution rates during turbidostat control were varied by a custom control loop and a proportional-integral-derivative (PID) controller to maintain an optical-density setpoint by varying the flow rate of medium through the reactor. Lighting for the photobioreactor is provided by 32 illuminators, 16 at 630 nm, and 16 at 680 nm. Additionally, six LI-COR Biosciences quantum sensors were used to measure light intensities within the photobioreactor, three sensors for measuring incident light, and three sensors for measuring transmitted light.

The light control software, Biolume, allowed for proportional-integral-derivative (PID) control of incident or transmitted light intensity. All cultures were pre-grown in turbidostat mode for at least 24 h using 54 μmol m^−2^ s^−1^ fixed incident irradiance (Melnicki et al., 2013).

### Turbidostat

A turbidostat was made by linking the voltage output of a transmitted light signal to the input of a BioFlo 310 controller. A custom loop and cascade control scheme was made to vary the flow rates of pumps to maintain a transmitted light voltage setpoint. The PID controller settings were tuned to allow fast but accurate control of the turbidity.

### Medium composition

A modified version of ASP2 medium (A+ Medium) was used, which contained the following components per 1 L: 0.70 g NaOH, 0.030 g Na_2_-EDTA, 0.60 g KCl, 0.27 g CaCl_2_•2H_2_O, 5.0 g MgSO_4_•7H_2_O, 1 mL of P1 Metals solution (0.55 mM H_3_BO_3_, 0.022 mM MnCl_2_•4H_2_O, 0.0023 mM ZnCl_2_, 50 μ M CoCl_2_•6H_2_O, 18 μ M Na_2_MoO_4_•2H_2_O, and 12 μ M CuSO_4_•5H_2_O), 0.050 g KH_2_PO_4_, 18 g NaCl, and 0.91 g NH_4_Cl for 17 mM NH_4_Cl, or adjusted to 0.9 mM for nitrogen limitation. NaHCO_3_ was added after autoclaving to a final concentration of 40 mM except for carbon limitation when 7.7 mM was used.

### Carbon limited steady state

For the C-limited steady state culture medium the NaHCO_3_ was reduced to 7.7 mM from 40 mM in the unlimited and N- limited cultures. The O.D. value (abs at 730 nm) was 0.36 at harvest.

### Nitrogen limited steady state

For the N-limited steady state culture medium, the NH_4_Cl was reduced to 0.90 mM from 17 mM in the unlimited and C-limited cultures. The O.D. value (abs at 730 nm) was 0.36 at harvest.

### Nutrient-replete (unlimited) steady state

Cells were grown in a turbidostat bioreactor. The O.D. (abs at 730 nm) was 0.18 at harvest.

### Cell harvest, redox probe labeling, LC-MS sample preparation and quantitative analysis for *in vitro* and *in vivo* proteomics probe-labeled samples and global unlabeled samples

For each culture condition 20 mL samples were collected in 50 mL falcon tubes. For each condition, four sample aliquots were placed in the dark for 3 h and an additional 3 “light” sample aliquots were irradiated for 3 h. Post incubation, all samples were spun down at 4500 × g for 10 min at 4°C. The “light” and “dark” samples were then decanted of media and the cell pellets were transferred with 500 uL PBS to 1.5 mL Eppendorf conical tubes. The probes were then added to the samples by addition of 1 uL each of 60 mM stock Mal-RP and IAM-RP. Probed samples were incubated in the dark for 60 min at 37°C with 250 rpm shaking. Next, samples were spun down at 4500 × g for 10 min at 4°C to pellet cells, and the supernatant was discarded. Also collected include two 20 mL sample aliquots from each nutrient condition, which were pelleted to be used for no probe control samples and another two 20 mL sample aliquots were pelleted for global proteome analysis. All cell pellets were then frozen in liquid nitrogen and stored till further sample preparation steps. Preparation for LC-MS analysis of probe-labeled and global samples, and quantitative data analysis was performed as described previously (Sadler et al., 2014). Details of LC-MS analysis are provided in the Supplemental Information text, and was performed as described previously (Sadler et al., 2014).

### Data availability

The mass spectrometry proteomics data have been deposited to the ProteomeXchange Consortium via the PRIDE partner repository with the dataset identifier PXD000897 and DOI 10.6019/PXD000897.

## Results and discussion

### *In vivo* chemical proteomics approach for analysis of redox-sensitive *Synechococcus* 7002 proteins

**Figure 1:**
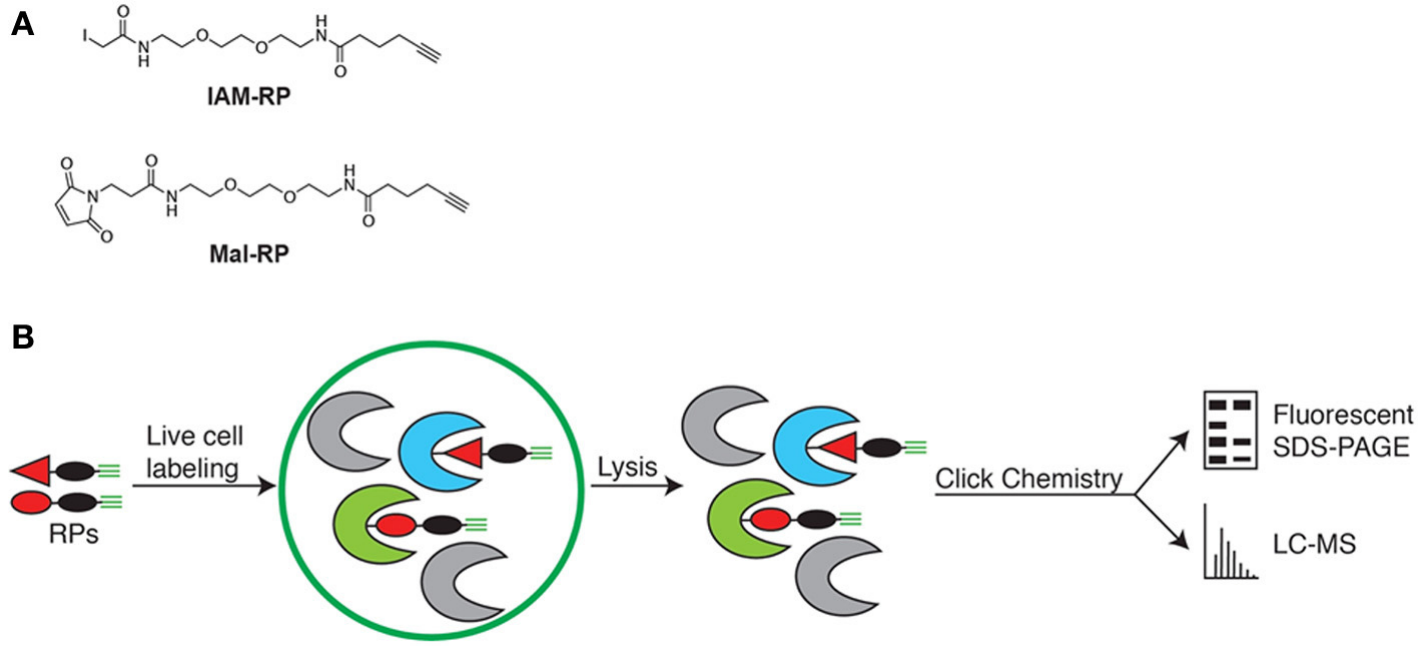
**(A)** Chemical probes for *in vivo* labeling of reduced cysteine thiols. IAM-RP and Mal-RP. **(B)** Schematic of *in vivo* chemical proteomics approach for analysis of redox-sensitive *Synechococcus* 7002 proteins.

Live cell measurements of redox state using chemical probes (Sadler et al., 2014) provide an alternative to more commonly employed *in vitro* methods (Lindahl et al., 2011), which less effectively capture true physiological redox states because lysis induces near complete oxidation of sensitive cysteine thiols thereby destroying the true cellular redox states of proteins (Hansen and Winther, 2009; Leonard and Carroll, 2011). Thus, to enable *in vivo* quantitative analysis of redox-sensitive *Synechococcus* 7002 proteins, we utilized a mix of two cell-permeable cysteine thiol reactive redox probes (RPs) (Figure 1). The probes have three features to enable *in vivo* quantitative analysis of redox sensitive proteins: (i) an electrophilic iodoacetamide (IAM) or N-ethylmaleimide (Mal) reactive group to label the reduced thiol form of cysteines, (ii) ethylene glycol spacers to impart cell permeability and (iii) an alkyne handle that allows for the Cu(I)-catalyzed azide-alkyne cycloaddition (CuAAC) of multifunctional tags for fluorescent detection and visualization of probe-labeled proteins and for biotin tagging and streptavidin enrichment of probe-labeled proteins (Speers and Cravatt, 2004; Sadler et al., 2014). Both the IAM-RP and Mal-RP readily enter intact *Synechococcus* 7002 cells, permeating both cytosol and membrane intracellular compartments (Sadler et al., 2014). Quantitative analysis used the label-free accurate mass and time (AMT) tag proteomics approach (Zimmer et al., 2006).

### Application of chemical probes for *in vivo* identification of redox-sensitive proteins

**Figure 2:**
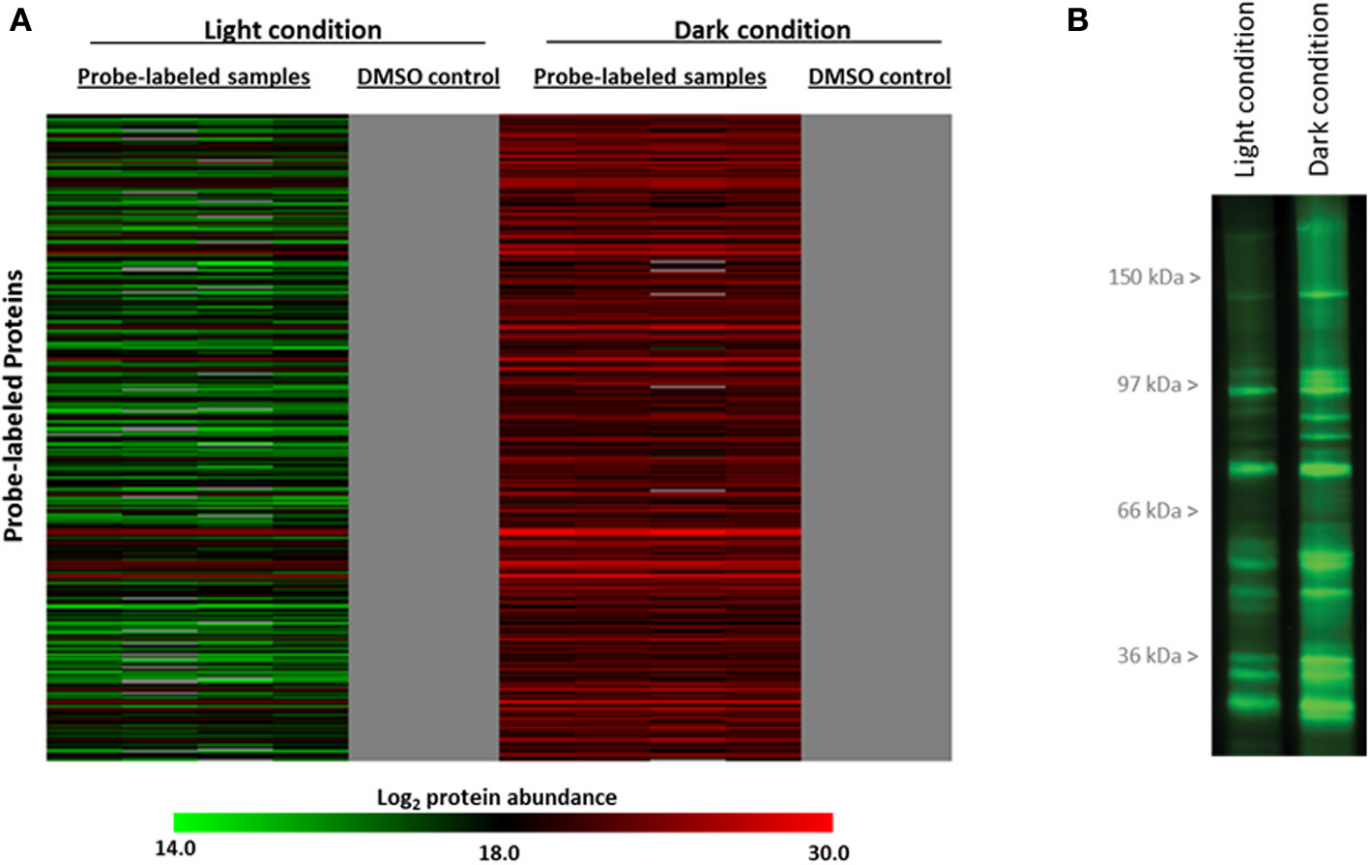
**(A)** Condition-dependent changes of *in vivo* labeling with IAM- and Mal-RPs in 300 redox-sensitive proteins identified. The heat map shows redox sensitive proteins exhibited increased probe labeling after incubation in the dark (red) relative to the light (green) condition in live-cell labeled *Synechococcus* 7002. Scale represents log2 of protein abundance and ranges from low (green) to high (red) protein abundance. The gray shading indicates protein not detected. Data in the heat map represent 4 replicates for probe-labeled samples and 2 replicates for control samples. **(B)** Complementary in-gel analysis of live-cell labeled *Synechococcus* 7002. Proteins were separated by SDS-PAGE and imaged by labeling with fluorescent probe tetramethylrhodamine-azide added by click chemistry.

To identify redox sensitive proteins *in vivo* and to evaluate redox control in the context of cellular redox state changes caused by the light to dark transition, the cell-permeable **IAM-RP** and **Mal-RP** were applied to two samples sets of live *Synechococcus* 7002 cells grown in continuous illumination (54 μmol m^−2^ s^−1^ fixed incident irradiance) under nutrient-replete conditions. The first set of cell samples were collected from the bioreactor and automatically probe labeled, while the second set of cells were transferred to a dark environment for 3 h prior to the addition of probe. To control for non-specific probe binding, we also analyzed DMSO-treated *Synechococcus* 7002 cells. These DMSO-treated “no probe” control samples specifically controlled for general non-specific binding during streptavidin-based enrichment. Excluding proteins that exhibited non-specific binding characteristics, LC-MS analyses across the probe-labeled light and dark samples identified a total of 350 proteins that were labeled by the cysteine thiol reactive redox probes. Thus, probe-labeled proteins that show significant quantitative difference between light and dark conditions (i.e., sensitive to light to dark transition) are identified as redox-sensitive proteins. We set the following criteria for designation of a protein as a redox-sensitive protein: (1) a significant difference across the probe-labeled light condition and the probe-labeled dark condition as judged by ANOVA (*p* < 0.05), (2) *a* ≥ 2-fold change in abundance in the probe-labeled dark condition relative to the probe-labeled light condition, (3) presence of at least one cysteine residue in candidate protein, and (4) reproducibility of peptide measurements across at least 3 of 4 probe-labeled sample replicates. Using the above conservative criteria, 300 proteins were designated as redox-sensitive proteins upon transition from light to dark in nutrient replete conditions (Supplementary Table 1). Strikingly, all 300 identified redox sensitive proteins exhibited increased probe labeling after incubation in the dark relative to the light condition indicating widespread protein reduction as result of the light-to-dark transition (Figure 2A). This was supported by in-gel analysis of live-cell labeled *Synechococcus* 7002, which showed much greater probe labeling in the dark (Figure 2B). The specific cysteines labeled by the redox probes were identified as described previously (Sadler et al., 2014), and are provided in Supplementary Table 1.

### Light-to-dark transitions reflect changes in protein redox state and not protein abundance

**Figure 3:**
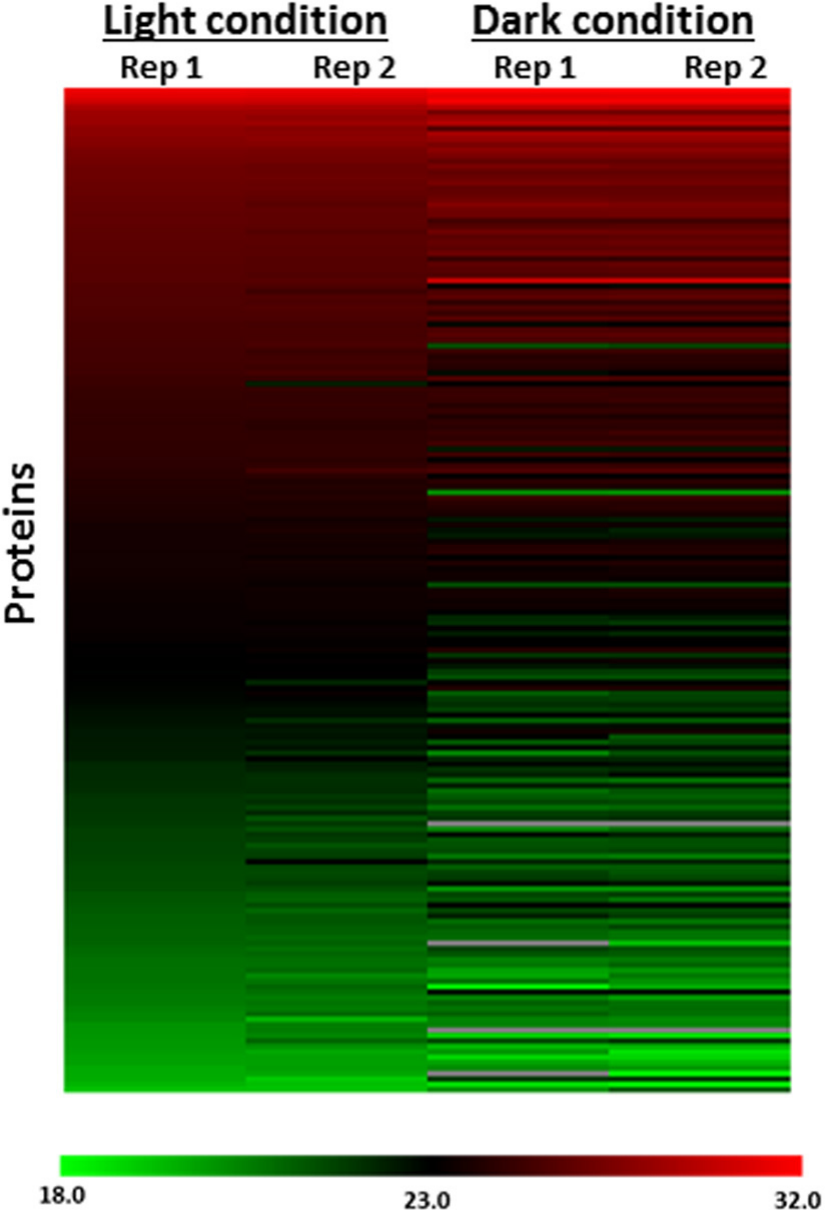
**Heat map representation of protein abundance profiles of 185 of 300 probe-labeled proteins designated as redox-sensitive identified in global (probe-free) LC-MS analysis**. The heat map shows increased probe labeling after incubation in the dark relative to the light condition in live-cell labeled *Synechococcus* 7002 exhibited by redox sensitive proteins is independent of protein abundance changes. Rep1, Replicate 1; Rep2, Replicate 2. Scale represents log2 of protein abundance and ranges from low (green) to high (red) protein abundance.

To determine if the differences measured between the light and dark conditions were due to changes in protein abundances, we performed global proteomics LC-MS analyses using unlabeled samples from the same conditions. A total of 514 proteins were identified in the global LC-MS analyses of unlabeled samples from the light and dark conditions. Of the 300 probe-labeled proteins designated as redox-sensitive, ~62% (185 proteins) were also identified in these global LC-MS analyses. This level of overlap results from the greater sensitivity achieved for lower level probe labeled proteins due to the finite dynamic range of the analyses. Importantly, only 7 of the 185 proteins (3.8%) showed any significant difference in protein abundance between the light and dark conditions (Figure 3), underscoring the fact that the differences observed between the light and dark conditions in the probe-labeled samples reflect changes in redox status that affect probe reactivity. Supplementary Figure 1 shows specific examples for the ribosomal protein RplV and the photosystem II protein PsbC.

### Confirmation and expansion of the cyanobacteria redox-sensitive protein knowledge base

Previously in the context of a nutrient limitation (carbon starvation) Sadler et al. reported identification of 176 redox sensitive proteins cycling in a dynamic fashion over a 60 min time continuum (Sadler et al., 2014). This single experiment yielded a greater than 2-fold increase in the number of recognized redox sensitive proteins in cyanobacteria combined from all prior reports (Lindahl and Florencio, 2003; Perez-Perez et al., 2006; Mata-Cabana et al., 2007; Lindahl and Kieselbach, 2009). We further expanded the number of known redox sensitive proteins to 300, which includes 137 of the 176 redox sensitive proteins (80%) described in that study also included in our list of 300 proteins reported as redox sensitive (see below and Supplementary Table 2). Thus, the present study effectively revealed an additional 163 redox-sensitive proteins in *Synechococcus* 7002 not previously appreciated.

Such a significant increase in the numbers of redox sensitive proteins reported in Sadler et al. (2014) and in the present study relative to earlier studies (Lindahl and Florencio, 2003; Perez-Perez et al., 2006; Mata-Cabana et al., 2007; Lindahl and Kieselbach, 2009) is likely a function of the analytical approach utilized. Both the current study and Sadler et al. employed a chemical proteomics approach that couples *in vivo* labeling of putative redox-controlled proteins using a cell-permeable cysteine thiol-specific redox probe with high resolution LC-MS analysis, while prior studies achieved measurements via *in vitro* methods including 2D gel and affinity column proteomic techniques. Nevertheless, a comparison of the 300 redox-sensitive proteins identified in the present study and the 69 redox controlled proteins identified in prior studies that did not utilize the analytical approach described above (Lindahl and Florencio, 2003; Perez-Perez et al., 2006; Mata-Cabana et al., 2007; Lindahl and Kieselbach, 2009) revealed a significant overlap. Fifty four of the 69 previously identified redox controlled proteins in *Synechocystis* PCC 6803 were identified in our list of 300 redox-sensitive proteins identified in *Synechococcus* 7002 further supporting the capability of our approach to both confirm and reveal new identifications (Supplementary Table 3).

### Functional analysis of *in-situ* labeled redox-sensitive proteins

The 300 redox-sensitive proteins identified in this study were partitioned by Cyanobase functional categories. All primary Cyanobase functional categories contained some redox-sensitive proteins, suggesting broad redox control of *Synechococcus* 7002 biological processes (Table 1 and Supplementary Table 1). Proteins involved in amino acid biosynthesis and nucleotide metabolism had the highest representation of any single functional category with 30% of proteins in each of these two functional categories determined to be redox sensitive. Transport/binding proteins represented the smallest functional category (1.3% of all proteins in this category) harboring three redox-sensitive proteins. Proteins annotated as hypothetical or unknown comprised 47 of the 300 redox-sensitive proteins (~16%), suggesting a large unexplored territory of redox-sensitive proteins and cyanobacterial redox biology.

**Table 1:** **Cyanobase functional categories represented by live-cell probe-labeled proteins identified during light-to-dark switch in *Synechococcus* 7002**.

	**300 redox sensitive protein**		**163 new redox sensitive proteins**		**Genome annotation**
**Cyanobase functional category**	**Protein count**	**Percent of gene count**	**Protein count**	**Percent of gene count**	**Gene count**
Amino acid biosynthesis	35	30	18	16	115
Biosynthesis of cofactors, Prosthetic groups, and carriers	30	22	18	13	139
Cell envelope	8	13	6	9	64
Cellular processes	9	9	7	7	101
Central intermediary metabolism	6	18	3	9	34
DNA replication, restriction, modification, recombination, and repair	4	4	4	4	89
Energy metabolism	21	22	14	15	95
Fatty acid, phospholipid, and sterol metabolism	12	26	7	15	46
Hypothetical	44	4	27	2	1169
Other categories	20	9	11	5	234
Photosynthesis and respiration	27	18	1	1	153
Purines, pyrimidines, nucleosides, and nucleotides	14	30	9	20	46
Regulatory functions	10	6	7	4	160
Transcription	5	14	2	6	35
Translation	49	24	24	12	201
Transport and binding proteins	3	1	2	1	234
Unknown	3	1	3	1	224
Total	300		163		3139

*The first column represents all redox sensitive proteins identified in this study. The second column represents novel redox sensitive proteins identified in this study. The third column represents all protein coding genes predicted from the genome annotation*.

A similar analysis of the subset of 163 newly reported redox sensitive proteins showed an almost identical trend; with proteins involved in amino acid biosynthesis and nucleotide metabolism having the highest representation of any single functional category, transport/binding and photosynthesis proteins representing the smallest functional category and hypothetical and unknown proteins comprising ~18% of the redox sensitive proteins (Table 1 and Supplementary Table 2). We note that the low representation of photosynthesis/respiration category is function of the known/expected role of redox control in photosynthesis/respiration wherein we would expect very few if no new redox sensitive proteins in this category beyond the ones previously reported. Taken together this observation suggests the subset of 163 newly reported redox sensitive proteins have no underlying functional differences. Thus, for the remainder of this work we focus the functional analysis on the aggregate set of 300 redox-sensitive proteins identified.

A striking observation from a comparative analysis of the 300 redox-sensitive proteins identified in the current study with the 176 redox-sensitive proteins recently described by Sadler et al. was the absence of any redox-sensitive proteins in the DNA metabolism and repair functional category in that study, while in the present study four DNA metabolism and repair proteins, MutM, GyrA, GyrB, and RecA were identified as redox sensitive. One simple explanation for the lack of detection in Sadler et al. could be that the decreased number of proteins identified in that study also reduces the likelihood of identifying proteins from more functional categories. However, a more likely explanation for the observed difference in labeling of DNA metabolism and repair proteins could be a function of the experimental conditions applied in each study. Sadler et al. (2014) performed their analysis on a very short timescale sampling over 60 min while in the current study sampling is performed on steady state cultures incorporating a 3 h incubation in light or dark; the increased timescales are more likely to capture events of cellular stress that often involve DNA replication and repair processes.

In agreement with prior observations, several proteins involved in photosynthesis and respiration were identified as redox sensitive in this study. These included three of the eleven PSI proteins, specifically the reaction center proteins PsaA and PsaB, as well as PsaD, which have recently been described as redox-sensitive PSI proteins in cyanobacteria (Lindahl and Kieselbach, 2009; Sadler et al., 2014). We also detected eight out of thirteen phycobillisome antenna complex proteins including apcABCDE and cpcABG. Additionally, two PSII proteins, the membrane spanning integral chlorophyll antenna proteins PsbB and PsbC, as well as PetH, commonly referred to as the ferredoxin NADP(H) reductase (FNR) and NADH dehydrogenase subunit NdhK were also detected.

A promising strategy to develop sustainable biofuels is the generation of fatty-acid-based biofuels produced by genetically engineered cyanobacteria (Ruffing, 2013). Previously only two fatty acid metabolism proteins had been reported as redox-regulated, acetyl CoA-carboxylase in spinach (Balmer et al., 2003) and malonyl-CoA:acyl-carrier-protein transacylase in chlamydomonas (Lemaire et al., 2004). Our results in *Synechococcus* 7002 suggest a much broader redox-control of fatty acid metabolism. We detected several important fatty acid biosynthesis proteins to be redox-controlled. These included AccABCD and components of the multi-subunit enzyme acetyl CoA-carboxylase, which catalyzes the first committed step of fatty acid biosynthesis. We also detected the β-ketoacyl-acyl carrier protein synthase III (FabH), 3-oxoacyl-(acyl carrier protein) synthase II (FabF), and NADH-dependent enoyl-[acyl-carrier-protein] reductase (FabI). FabH is an essential enzyme that conducts the major condensation reaction in the initiation of type II fatty acid biosynthesis in both Gram-positive and Gram-negative bacteria (Lai and Cronan, 2003). FabF works in concert with FabH to catalyze the condensation reaction of fatty-acid synthesis by the transfer of two C atoms from malonyl-ACP to an acyl acceptor (Rock and Jackowski, 2002). FabI is a key enzyme in type II fatty-acid synthases that catalyzes the last step in each elongation cycle (Heath et al., 2000).

While redox control of numerous transcription factors has been shown in eukaryotic organisms (Bloomfield et al., 2003; Funato et al., 2006; Fleming et al., 2009; Tell et al., 2009; de Keizer et al., 2011), it is only recently being appreciated in cyanobacteria (Sadler et al., 2014). In further support of this notion, our data revealed eight redox-sensitive transcriptional factors including three, RbcR, Zur, and SYNPCC7002_A2523, that have been recently reported (Sadler et al., 2014). Among the five newly identified redox-sensitive transcription factors reported in this study were PedR, RpaB. PixL, PixG, and SYNPCC7002_A1955. The photosynthetic activity responsive transcriptional regulator, PedR, was shown to interact with thioredoxin, and subsequent reduction of PedR by thioredoxin causes transient inactivation of PedR upon light perturbation of cyanobacterial cells (Horiuchi et al., 2010). Consistent with this prior observation PedR was identified in our analysis as a redox-sensitive transcriptional regulator. PixL and PixG form the PixL-PixGH two-component regulatory system involved in positive control of phototaxis in *Synechocystis* 6803 (Bhaya, 2004). Although *Synechococcus* 7002 is not apparently motile, the model cyanobacterium *Synechocystis* 6803 can move on solid surfaces toward light by twitching motility (Choi et al., 1999; Yoshihara and Ikeuchi, 2004). The apparent redox-sensitivity of this two component system regulator suggests it warrants further study. RpaB is the two component signal transduction system, response regulator of photosystem I and represents the second of three two component system regulators newly identified as redox-sensitive in this study. Given the acknowledged redox control of photosynthesis this is not unexpected and suggests a complex regulatory system where coupled redox and translational control of the RpaB regulon in part mediates cyanobacteria response to environmental transitions. SYNPCC7002_ A1955 is annotated as a LuxR family two component signal transduction system response regulator similar to SYNPCC7002_A2523 which has also been recently reported as a redox sensitive transcriptional regulator (Sadler et al., 2014).

### Effect of nutrient limitation on cellular redox state

In order to distinguish between the effects of growth rate upon redox status from those due to the nature of growth limitation, *Synechococcus* 7002 was grown at the same rate in chemostat cultures where the limiting nutrient was C(NaHCO_3_ and CO_2_), or N (NH_3_). Samples collected from steady-state cultures were then incubated in either the light or the dark and then labeled with **IAM-RP** and **Mal-RP** *in vivo*.

**Figure 4:**
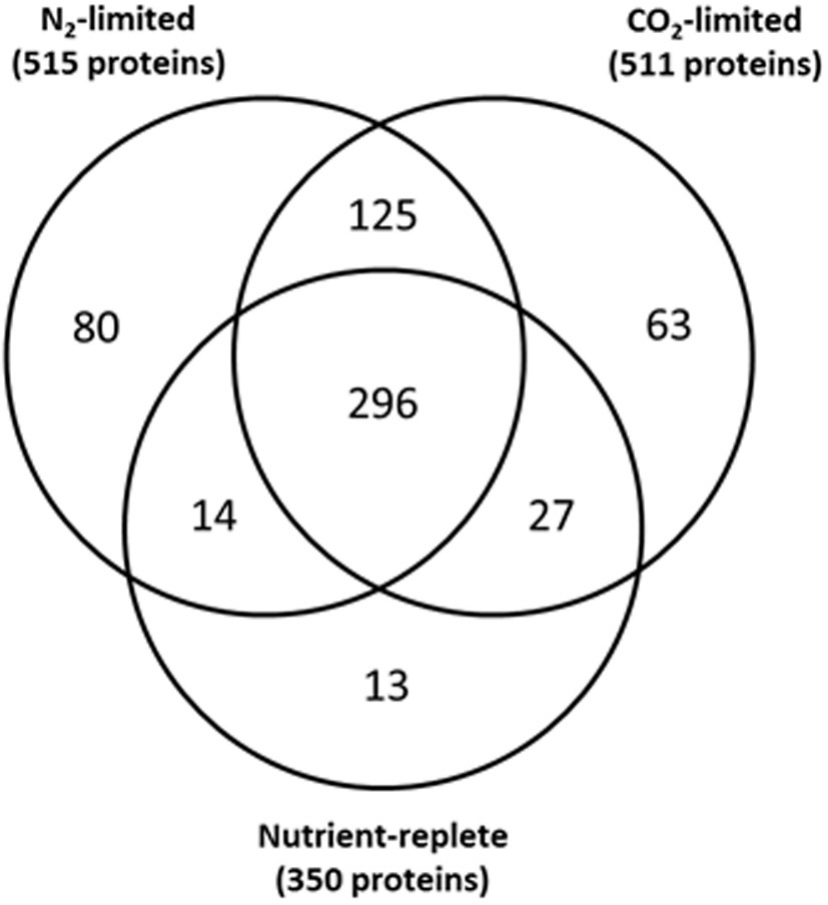
**Overlap of probe labeled proteins under nutrient-replete conditions, C-limited conditions, and N-limited conditions**.

LC-MS analyses of C- or N-limited probe-labeled *Synechococcus* 7002 samples resulted in the confident identification of 511 and 515 proteins for the C-limited and N-limited samples, respectively. The number of labeled proteins under these nutrient-limited conditions was approximately 50% higher than in nutrient replete cultures (Figure 4 and Supplementary Table 1, Sheets 1, 3, and 5). Interestingly, complementary global LC-MS analyses of unlabeled nutrient replete, C-limited, and N-limited samples showed essentially equivalent protein identifications; with nutrient replete cultures having 4% more protein identifications than C-limited cultures and only 10% less identifications than N-limited cultures (Supplementary Table 4). Taken together, these results suggest that N- or C-limitation results in increased probe labeling of proteins than in nutrient replete samples, indicative of a more reduced cellular environment. This phenomenon can be explained by previous studies which show that in the absence of downstream electron acceptors (N and CO_2_) plastoquinone (PQ) and NADP+ become overly reduced (Miller and Canvin, 1989; Hauf et al., 2013; Salomon et al., 2013; Osanai et al., 2014); our data shows that this overreduction is extended to the proteome.

All but 13 of the 350 proteins labeled under the nutrient replete condition (4%) were also labeled either under C- or N-limitation (Figure 4 and Supplementary Table 5), with most of these proteins uniquely labeled in nutrient replete conditions annotated as hypothetical proteins. Examination of global proteomics data for N-limited and C-limited conditions showed that only 4 of the 13 proteins uniquely labeled in nutrient replete conditions were also synthesized under N-limited and C-limited conditions suggesting the unique presence of the majority of proteins in the nutrient replete cultures may be a function of their activity under those conditions (Supplementary Table 5). Eighty proteins and 63 proteins were uniquely labeled under N-limitation and under C-limitation, respectively (Supplementary Table 5). Of the 80 proteins singularly unique under N-limitation, only ~30% were synthesized under nutrient replete and C-limited conditions as determined by global proteomics data. Additionally, only 11 of the 63 proteins (~17%) uniquely labeled in C-limited conditions were synthesized under nutrient replete and N-limited (Supplementary Table 5). These results also suggest that the unique presence of the overwhelming majority of these proteins is likely a function of their activity under that specific condition.

As specified previously, we identified condition-specific probe labeled proteins. In the catalog of 80 unique proteins identified in N-limitation, many were involved in N-scavenging and catabolic processes, including UrtA, UrtD, and UrtE, all of which are ABC-type, high affinity urea transporters (Valladares et al., 2002). Cyanate hydratase (CynS) was detected as well and is part of the cynABDS operon which is responsible for converting cyanate to carbon dioxide and ammonia in a bicarbonate-dependent reaction. CynS also serves in the detoxification of cyanate resulting from intracellular urea decomposition (Kamennaya and Post, 2011). Cyanobacteria can store N as cyanophycin (a polymer of aspartate and arginine) under N-excess conditions, and utilize the polymer when N is limiting. We labeled L-arginine deiminase (ArcA) which converts arginine into citruline and NH_3_. We also labeled potential arginase/agmatinase type enzymes, which participate in the metabolism of amino groups.

Additionally, probe labeled proteins specific to the C-limited samples include BicA, a bicarbonate transporter, and CupA, which is involved in C concentrating and is induced under low C conditions (Shibata et al., 2001). In addition, the transcriptional regulator of high-affinity CO_2_ uptake, CcmR was labeled only in the C-limited condition. Furthermore, NAD(P)H dehydrogenase 1(NDH-1) subunits I and A were under controlled under C-limited conditions. NDH-1 is essential for both CO_2_ uptake and cyclic electron transport (Shibata et al., 2001).

### Impact of nutrient limitation on protein redox dynamics

**Figure 5:**
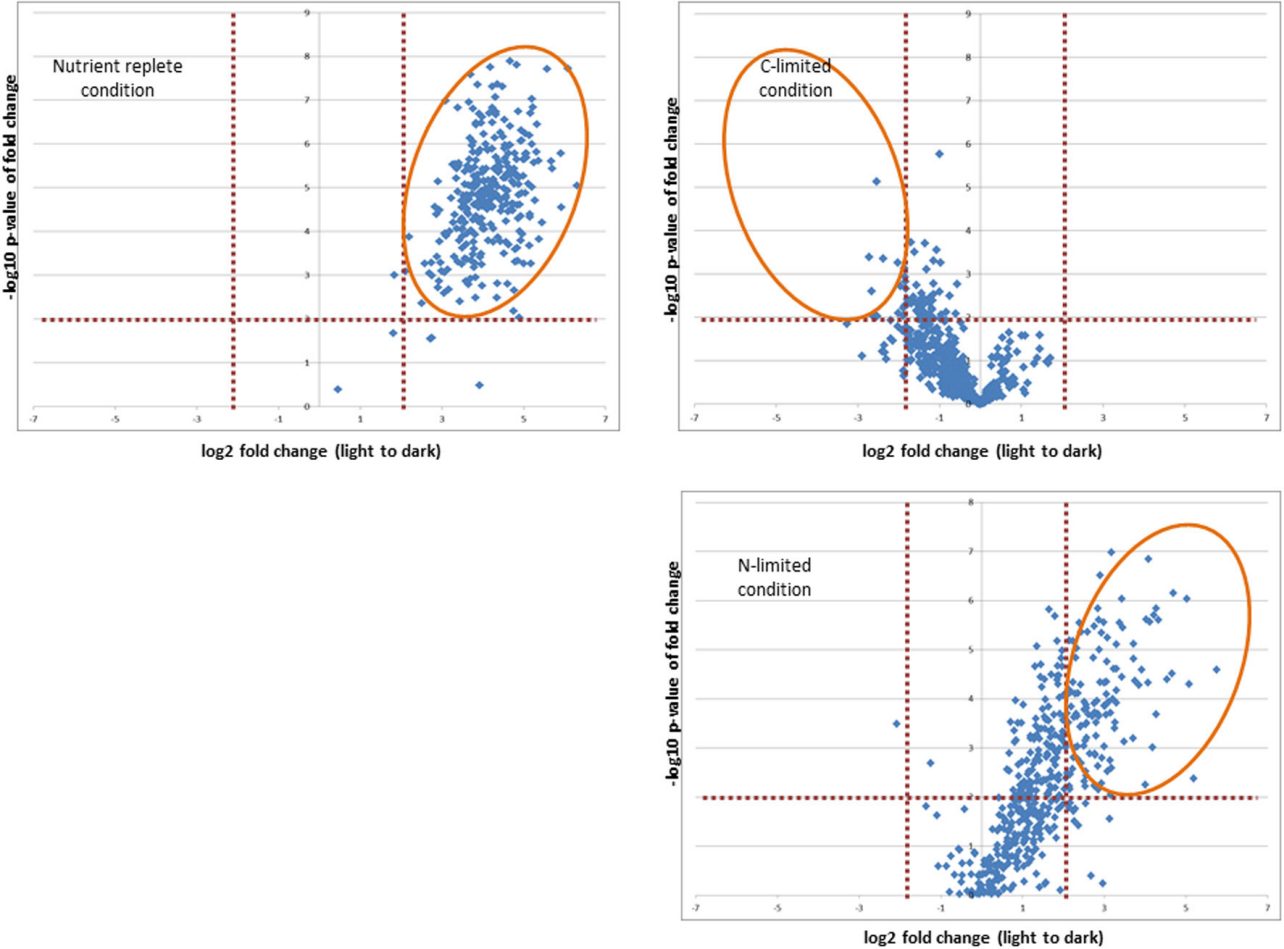
**Effect of shift from light to dark to on the capacity for reductant transfer to proteins under each nutrient limitation**. Orange elipticals highlight significantly changing proteins (*p*-values < 0.01 and log2 fold change >2 or < −2).

In addition to comparing redox status between different resource limitations, we examined the effect of a shift from light to dark to evaluate the capacity for reductant transfer to proteins under each nutrient limitation. Our analysis was restricted to those proteins which our conservative criteria deemed redox sensitive. Whereas 300 proteins significantly altered redox status in the culture whose starting point was most oxidized (nutrient replete), only 10 proteins shifted in the steady-state culture that was most reduced (C-limited) while the N-limited cultures exhibited an intermediate effect, with 142 proteins showing a significant quantitative difference in response to the light-to-dark transition (Figure 5 and Supplementary Table 1, Sheets 2, 4, and 6). The overwhelming majority of the redox sensitive proteins identified are part of the “common or semi-common core” per the Venn diagram in Figure 4, with very few uniquely labeled for a specific condition. For example, only 5 of the 300 redox sensitive proteins under nutrient replete conditions are uniquely labeled for that condition. The striking observation that only 10 proteins showed a significant quantitative difference in response to the light-to-dark transition for the C-limited culture, a 30-fold decrease compared to the proteins that showed a significant quantitative difference in response to the light-to-dark transition for the nutrient-replete culture (300 proteins), suggests C-limitation results in such a significant over-reduction of the system under light conditions that the transition to dark has a negligible effect on redox status of the cell, hence the smaller number of significantly different proteins. Importantly this result is consistent with the hypothesis that CO_2_ serves as a major sink for reductants generated during the photosynthesis and electron transport process and that absence or limitation of this important nutrient result in significant over-reduction of redox-sensitive proteoforms. While this result might have been anticipated, it is important to note that this work is the first to provide a relative quantitative measure within live cells.

## Conclusion

Post-translational modifications (PTMs) in proteins provide a mechanism to rapidly regulate catalytic activity, and oxidation-reduction reactions at cysteine residues are a known mechanism. To interrogate the dynamic nature of the changes in redox status in *Synechococcus* 7002, we applied cysteine thiol-specific probes to which cells are permeable and hence permit identification of redox sensitive proteins *in vivo*. We showed that this approach can detect redox changes in proteins involved in all major biological processes in *Synechococcus* 7002, including proteins involved in energy metabolism, translation/protein synthesis and photosynthesis and respiration, confirming and extending our prior observations (Sadler et al., 2014). We also observed that the number of labeled proteins under N- or C-limitation was approximately 50% greater than in nutrient replete cultures, suggesting that N- or C-limitation results in increased probe labeling of proteins than in nutrient replete samples, indicative of a more reduced cellular environment. Furthermore, our results suggest dynamic redox changes in response to specific nutrient limitations contribute to the regulatory changes being driven by a shift from light to dark.

Current and future algae and cyanobacteria studies have and will continue to engineer genetic mutants to enhance the production of specific biofuel precursors and products. However, an oversight in this approach is that PTM's play a significant role in metabolic processes. Identifying PTM enzyme/substrate partners and the molecules involved in signal transduction must be weaved into mutant studies in order to optimize the phototroph's physiology and subsequent desirable hydrocarbon output. Our approach is to identify which proteins are redox sensitive as a preliminary step, while follow up studies could include our results to match redox partners and their roles in metabolism in order to optimize their proposed mutants. An example of a metabolic process that is well known to be redox regulated is the Calvin-Benson cycle. We identified 9 of the 11 CBC enzymes in our experiment (and also identified these enzymes in our initial paper Sadler et al., 2014). However, we desired to identify other novel pathways that rely on redox reactions to function appropriately. We delineate how redox regulation is crucial to many key biosynthetic pathways by characterizing a broad range of enzymes involved in multiple biosynthetic processes that were probe labeled in our study. Above we mention the current research in cyanobacteria mutants to induce the overexpression of enzymes involved in fatty acid and lipid synthesis. Our identification of redox sensitive enzymes involved in these processes can potentially enrich the experimental design of research in the field biofuel production.

### Conflict of interest statement

The authors declare that the research was conducted in the absence of any commercial or financial relationships that could be construed as a potential conflict of interest.
