# Supplementary material for: Characterization of protein redox dynamics induced during light-to-dark transitions and nutrient limitation in cyanobacteria
Source: Front Microbiol. 2014 Jul 3;5:325. doi: 10.3389/fmicb.2014.00325 (PMC4080843; doi:10.3389/fmicb.2014.00325)
Supplement: Supplementary file 1 [file Presentation1.ZIP › Supplemental Data.docx]

**SUPPLEMENTAL DATA**

**Figure Legend**

Supplemental Figure 1. Probe reactivity and protein abundance profile for the proteins RplV and PsbC showing increased probe labeling after incubation in the dark relative to the light condition in live-cell labeled *Synechococcus* 7002 while protein abundance for both RplV and PsbC decreases.

**Experimental Procedures**

**LC-MS Analysis of Probe-Labeled and Global Unlabeled Proteomics Samples Utilizing AMT tag Approach**

In brief, this approach utilizes tandem mass spectrometry to generate a reference peptide database (accurate mass and time tag database; AMT tag database) of observed peptides, their associated theoretical masses, and LC elution times (normalized). This database is utilized to assign peptide sequences to ion current (relative abundance) information of peptides measured using high-resolution, high mass measurement accuracy mass spectrometry (LC-MS). As part of this approach, no-probe control samples, probe-labeled samples, and global proteomics samples for LC-MS analysis were analyzed using an LTQ-Orbitrap Velos™ (ThermoFisher Scientific, San Jose, CA) mass spectrometer interfaced with a reverse phase HPLC system for peptide separation (LC-MS). Peptides were reverse-phase separated on in-house manufactured columns (60 cm × 360 µm o.d. × 75 µm i.d. fused silica capillary tubing) packed with 3 µm Jupiter C_18_ stationary phase (Phenomenex, Torrence, CA). The HPLC system was equilibrated with 100% mobile phase A (0.1% formic acid in water). Fifty minutes after peptide injection, mobile phase B (0.1% formic acid acetonitrile) displaced mobile phase A, generating an approximate exponential gradient. Split flow controlled the gradient speed operating under constant pressure (10 kpsi). Separated peptides were ionized (positive) using an electrospray ionization interface (manufactured in-house) that consisted of chemically etched electrospray emitters (150  µm o.d. 20  µm i.d) *^(^*[*^1^*](#_ENREF_1)*^)^*. The LTQ-Orbitrap Velos™ MS was operated using a heated capillary temperature and spray voltage of 200°C and 2.2 kV, respectively. Data was acquired for 100 minutes, beginning 65 minutes after sample injection (15 minutes into gradient). Orbitrap™ spectra were collected from 400–2000 m/z at a resolution of 100k followed by data-dependent ion trap generation of MS/MS spectra of the six most abundant ions using a collision energy of 35%. A dynamic exclusion time of 30 seconds was used to discriminate against previously analyzed ions.

Generated MS/MS spectra were searched using the SEQUEST algorithm (V27, revision 12) *^(^*[*^2^*](#_ENREF_2)*^)^* against the publicly available *Synechococcus* 7002 translated genome sequence, and re-scored using the MS-GF approach *^(^*[*^3^*](#_ENREF_3)*^)^*. Identified peptides of at least six amino acids in length having MS-GF score ≤1E-10, which corresponds to an estimated FDR <1% at the peptide level, were used to generate an AMT tag database. This database includes LC-MS measurements from probe-labeled samples, and the global proteomic analyses.

Orbitrap™ spectra were deisotoped using the software tool Decon2LS *^(^*[*^4^*](#_ENREF_4)*^)^*, after which mass and elution time features were identified and matched with VIPER *^(^*[*^5^*](#_ENREF_5)*^)^* to peptides stored in the *Synechococcus 7002* AMT tag database within mass measurement accuracy and elution time accuracy cut-offs of <2 ppm and <2%, respectively. Measured arbitrary abundance for a particular peptide was determined by integrating the area under each LC–MS peak for the detected feature matching to that peptide. Matched features from each Orbitrap™ analysis (dataset) were then filtered on a false discovery rate (FDR) of less than or equal to 5%; the FDR associated with the AMT tag proteomics approach is calculated using STAC (Statistical Tools for AMT tag confidence), a statistical algorithm for assigning confidence to matched mass and elution time features *^(^*[*^6^*](#_ENREF_6)*^)^*.

Relative peptide abundance measurements in technical replicates were scaled and normalized using linear regression in DAnTE *^(^*[*^7^*](#_ENREF_7)*^)^*. Normalized peptide abundance values were then rolled up to proteins using RRollup *^(^*[*^7^*](#_ENREF_7)*^)^*; a minimum of five peptides was required for the Grubb's test, with a p-value cutoff of 0.05. Only peptides unique in identifying a single protein were utilized to estimate protein abundances. Additionally, proteins represented by <2 unique peptides were removed. ANOVA analyses were applied to protein abundance data sets (p-value ≤0.05) to identify statistically significant differences in protein expression levels.

References

1. Kelly, R. T., Page, J. S., Luo, Q., Moore, R. J., Orton, D. J., Tang, K., and Smith, R. D. (2006) Chemically etched open tubular and monolithic emitters for nanoelectrospray ionization mass spectrometry, *Anal Chem* *78*, 7796-7801.

2. Yates, J. R., 3rd, Eng, J. K., McCormack, A. L., and Schieltz, D. (1995) Method to correlate tandem mass spectra of modified peptides to amino acid sequences in the protein database, *Anal Chem* *67*, 1426-1436.

3. Kim, S., Gupta, N., and Pevzner, P. A. (2008) Spectral probabilities and generating functions of tandem mass spectra: a strike against decoy databases, *J Proteome Res* *7*, 3354-3363.

4. Jaitly, N., Mayampurath, A., Littlefield, K., Adkins, J. N., Anderson, G. A., and Smith, R. D. (2009) Decon2LS: An open-source software package for automated processing and visualization of high resolution mass spectrometry data, *BMC Bioinformatics* *10*, 87.

5. Monroe, M. E., Tolic, N., Jaitly, N., Shaw, J. L., Adkins, J. N., and Smith, R. D. (2007) VIPER: an advanced software package to support high-throughput LC-MS peptide identification, *Bioinformatics* *23*, 2021-2023.

6. Stanley, J. R., Adkins, J. N., Slysz, G. W., Monroe, M. E., Purvine, S. O., Karpievitch, Y. V., Anderson, G. A., Smith, R. D., and Dabney, A. R. (2011) A statistical method for assessing peptide identification confidence in accurate mass and time tag proteomics, *Anal Chem* *83*, 6135-6140.

7. Polpitiya, A. D., Qian, W. J., Jaitly, N., Petyuk, V. A., Adkins, J. N., Camp, D. G., 2nd, Anderson, G. A., and Smith, R. D. (2008) DAnTE: a statistical tool for quantitative analysis of -omics data, *Bioinformatics* *24*, 1556-1558.
