# Supplementary figures and images for: Characterization of protein redox dynamics induced during light-to-dark transitions and nutrient limitation in cyanobacteria
Source: Front Microbiol. 2014 Jul 3;5:325. doi: 10.3389/fmicb.2014.00325 (PMC4080843; doi:10.3389/fmicb.2014.00325)

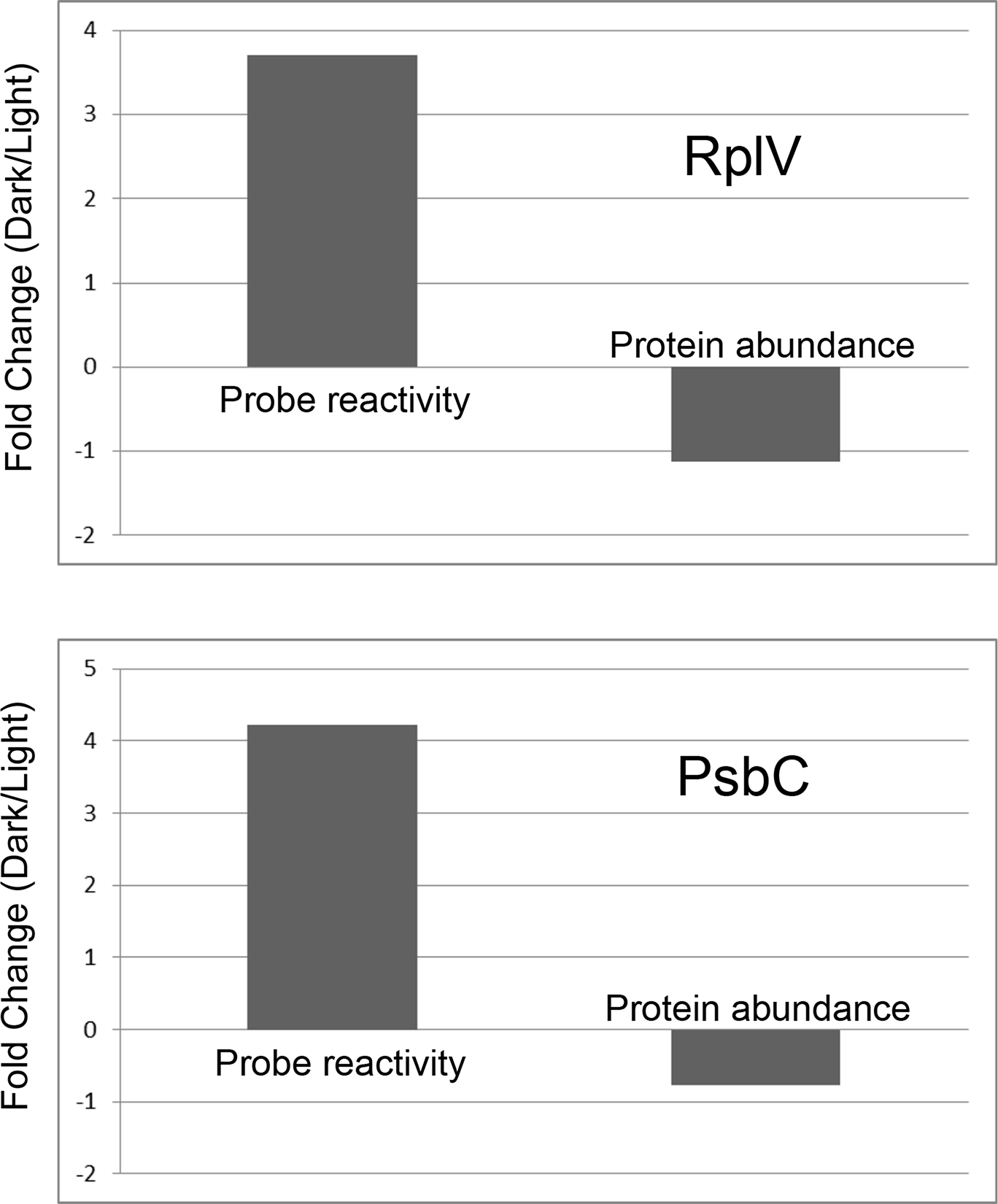

Supplement: Supplementary file 1 [file Presentation1.ZIP › Supplementary Figure 1.tif]
